# Pediatric dumbbell-shaped orbital schwannoma with extension to the cranial cavity: A case report and literature review

**DOI:** 10.3389/fneur.2022.1071632

**Published:** 2023-01-10

**Authors:** Yongjia Shao, Qian Xi, Ailan Cheng, Minghui Qian, Shuguang Chu

**Affiliations:** ^1^Department of Radiology, Shanghai East Hospital, Tongji University School of Medicine, Shanghai, China; ^2^College of International Studies, Yangzhou University, Yangzhou, Jiangsu, China

**Keywords:** children, cranial-orbital, dumbbell-shaped, schwannoma, full-cut

## Abstract

Orbital schwannomas are rare in children, especially those with intracranial extension. Herein, our report refers to a 12-year-old boy who had a cranial-orbital mass with a dumbbell-like appearance. The total neoplasms was successfully removed *via* a transcranial approach, and the pathological diagnostic result was schwannoma. Neither radiotherapy nor chemotherapy was performed after surgery, and no recurrences were observed for 3 months. Our report suggests that orbital schwannomas should be differentiated from other types of orbital tumors with sufficient evidence and that complete surgical resection remains the first choice to cure this disease.

## 1. Background

Intracranial schwannomas are common and account for 8–10% of all primary intracranial tumors (1). In contrast, orbital schwannomas are rare and account for only approximately 1–2% of all primary schwannomas (2). In this study, we report a case of schwannoma in children, which is a dumbbell-shaped cranial-orbital mass. The total neoplasm was successfully removed, and the pathological diagnostic result was schwannoma. Postoperatively, the patient's painless exophthalmos had completely disappeared without postoperative sequelae or visual impairment. Neither radiotherapy nor chemotherapy was performed, and no recurrences were observed for 3 months.

## 2. Clinical presentation

### 2.1. History

Our case refers to a 12-year-old boy with painless exophthalmos in the left eye. He had no relevant clinical symptoms, a previous medical history of binocular vision, and a family history of neurofibromatosis. Physical examination showed that binocular vision was normal, the eyeballs could move freely in all directions, and the eyes were sensitive to light reflection. In addition, there were no facial paresthesias or abnormal facial expression.

### 2.2. Imaging findings

**Figure 1:**
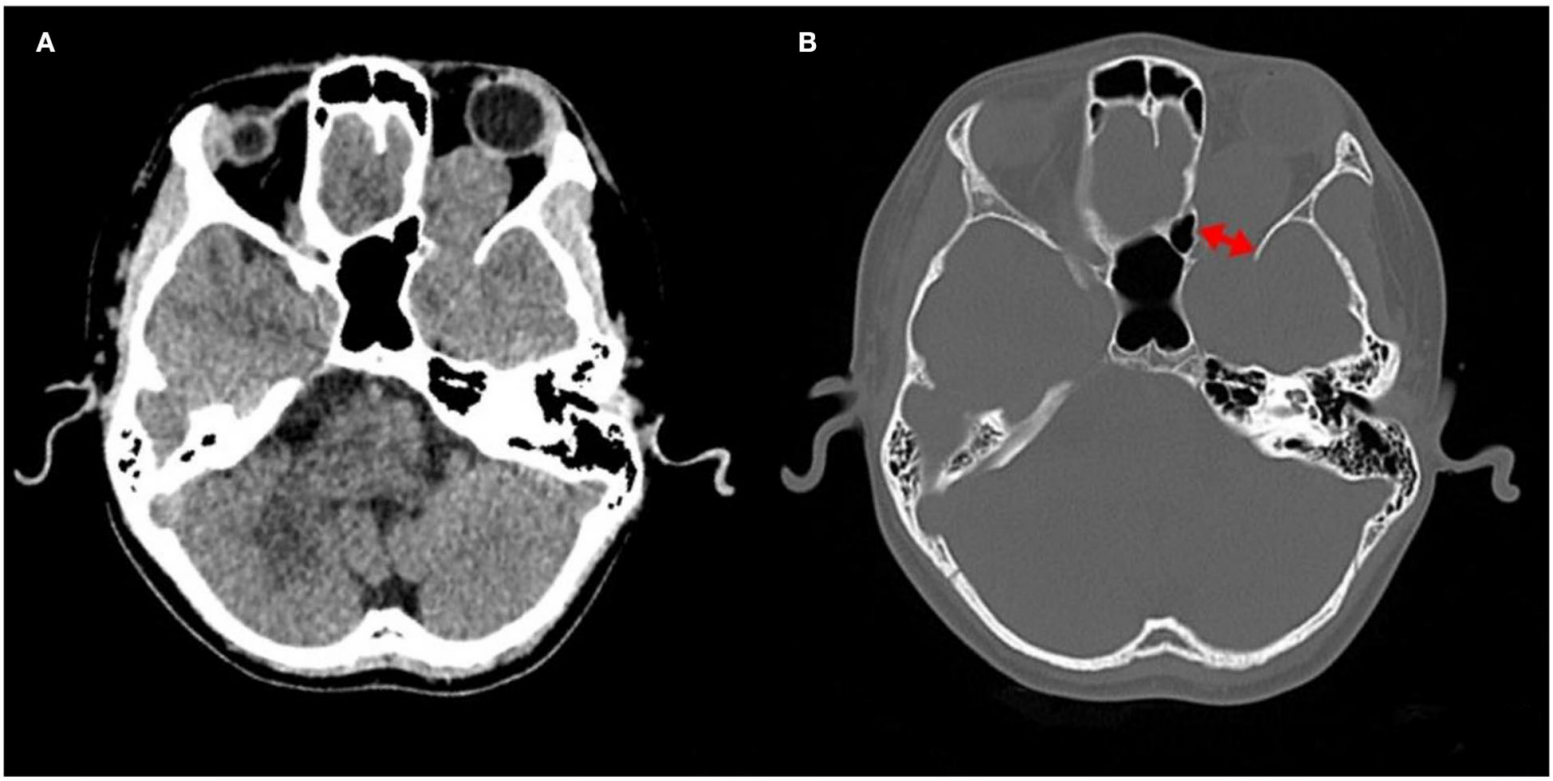
Images of computed tomography (CT). **(A)** CT revealing a mass in orbit, some of which seem to extend into the cranial cavity. **(B)** The bone window showing compressive displacement of the adjacent bone and widened supraorbital fissure (two-way arrow).

**Figure 2:**
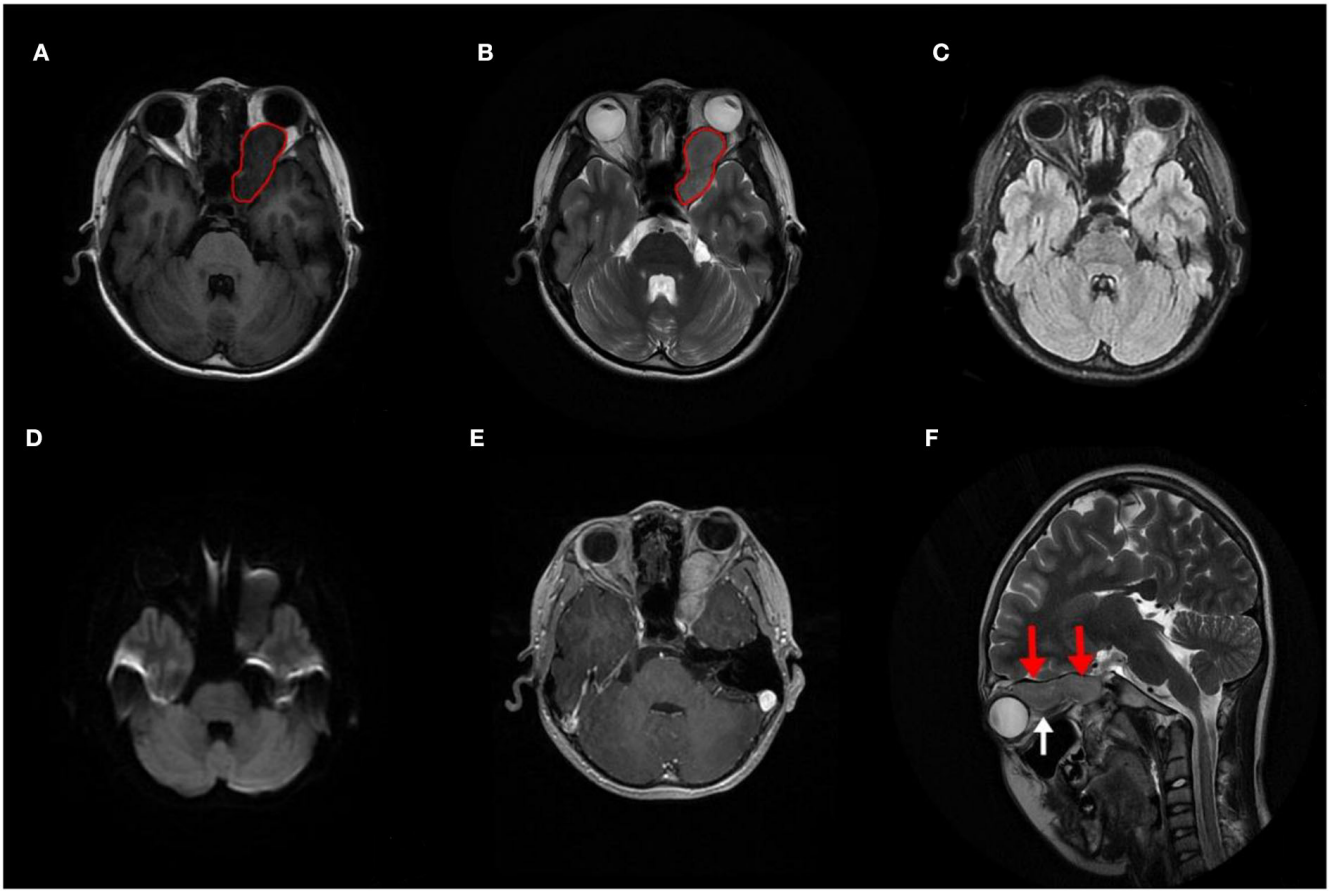
Preoperative image of magnetic resonance imaging (MRI). **(A–D)** MRI demonstrating a circumscribed mass that extended into the left cavernous sinus (red circle), with a slightly inhomogeneous hypointense signal on T1WI and a slightly inhomogeneous hyperintense signal on T2WI. Meanwhile, it shows an inhomogeneous isointense signal on fluid-attenuated inversion recovery (FLAIR) and a hypointense signal on diffusion-weighted imaging (DWI). **(E)** The mass showed clear and mild inhomogeneous enhancement after Gd injection. **(F)** The mass located above the optic nerve (red arrows), which was intact and not pressed (white arrow).

**Figure 3:**
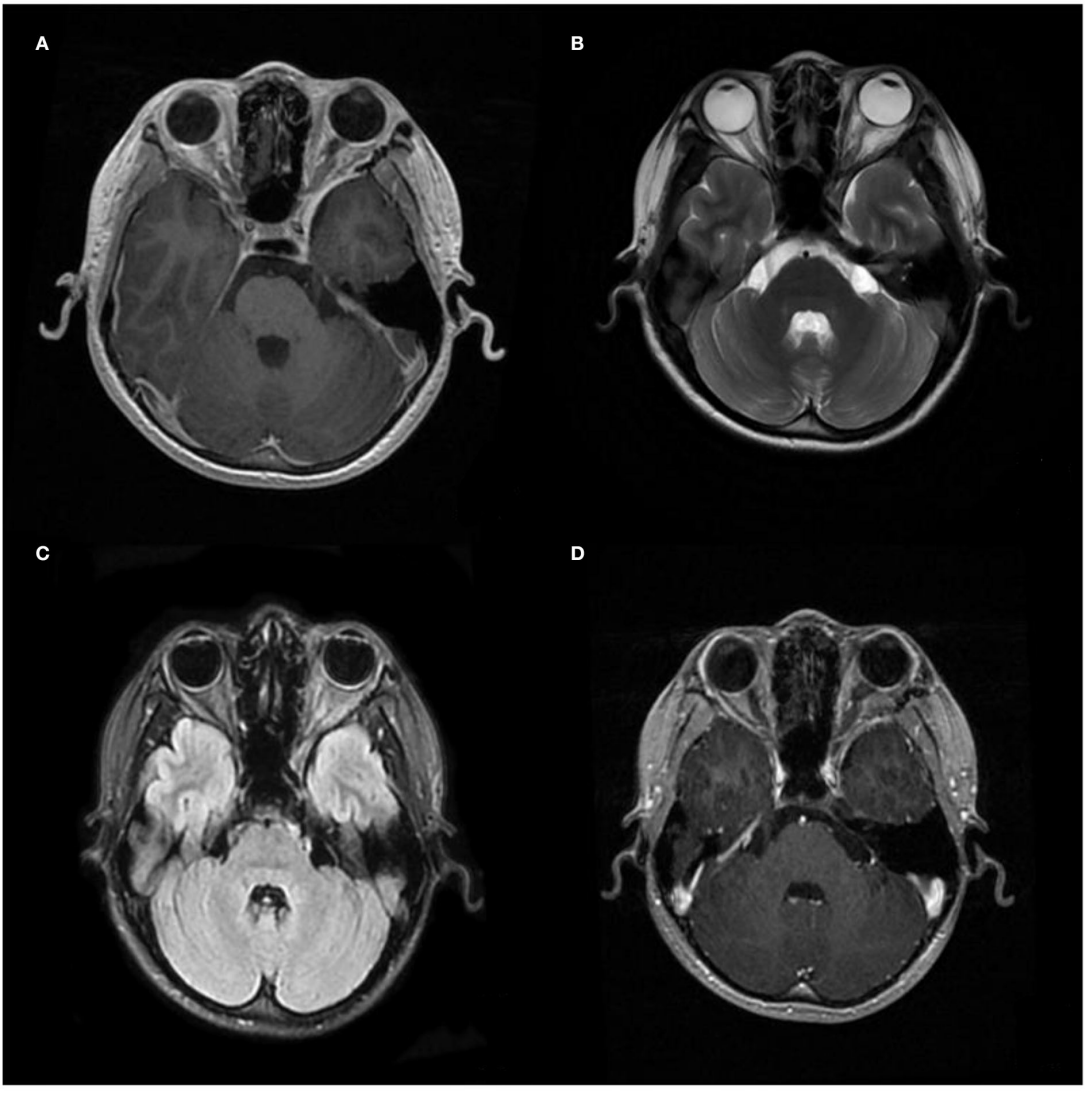
Postoperative image of MRI. **(A–C)** Images of T1WI, T2WI, and Flair, and **(D)** images after gadolinium (Gd) injection were performed 3 months after the operation, respectively, showing no recurrence.

A computed tomography (CT) scan revealed a homogeneous and non-calcified mass in the extraconal compartment of the left posterior eyeball that was similar in density to the brain parenchyma. The inner bone wall of the left orbit was thinner than before, with compressive displacement of the adjacent bone and widening of the supraorbital fissure (Figure 1). Magnetic resonance imaging (MRI) demonstrated an elliptical, well-defined, and homogeneous retrobulbar mass, including the left orbit, the orbital apex, and even the left cavernous sinus. The superior orbital rectus muscle was compressed and transformed; the optic foramen was enlarged, but the optic nerve was not obviously pressed. The mass presented as a slightly uneven hypointense signal, a hyperintense signal, and an isointense signal on T1-weighted images (T1WI), T2-weighted images (T2WI), and fluid-attenuated inversion recovery (FLAIR), respectively. Meanwhile, the mass showed a hypointense signal on diffusion-weighted imaging (DWI) and a low value of apparent diffusion coefficient (ADC), with apparent and inhomogeneous enhancement after gadolinium (Gd) injection (Figure 2). An MRI performed 3 months after surgery showed no tumor persistence (Figure 3).

### 2.3. Intraoperative findings and post-operative course

A gross total excision was performed *via* a left frontotemporal craniotomy. The solid tumor was found to be located above the eyeball and the optic nerve, which occupied the middle and posterior parts of the orbital region and extended to the anterior part of the cavernous sinus through the enlarged supraorbital fissure. The result of the intraoperative pathological diagnosis was a spindle cell tumor. Postoperatively, the patient's painless exophthalmos had completely disappeared, with no evidence of postoperative sequelae or visual impairment during a follow-up.

### 2.4. Pathological findings

**Figure 4:**
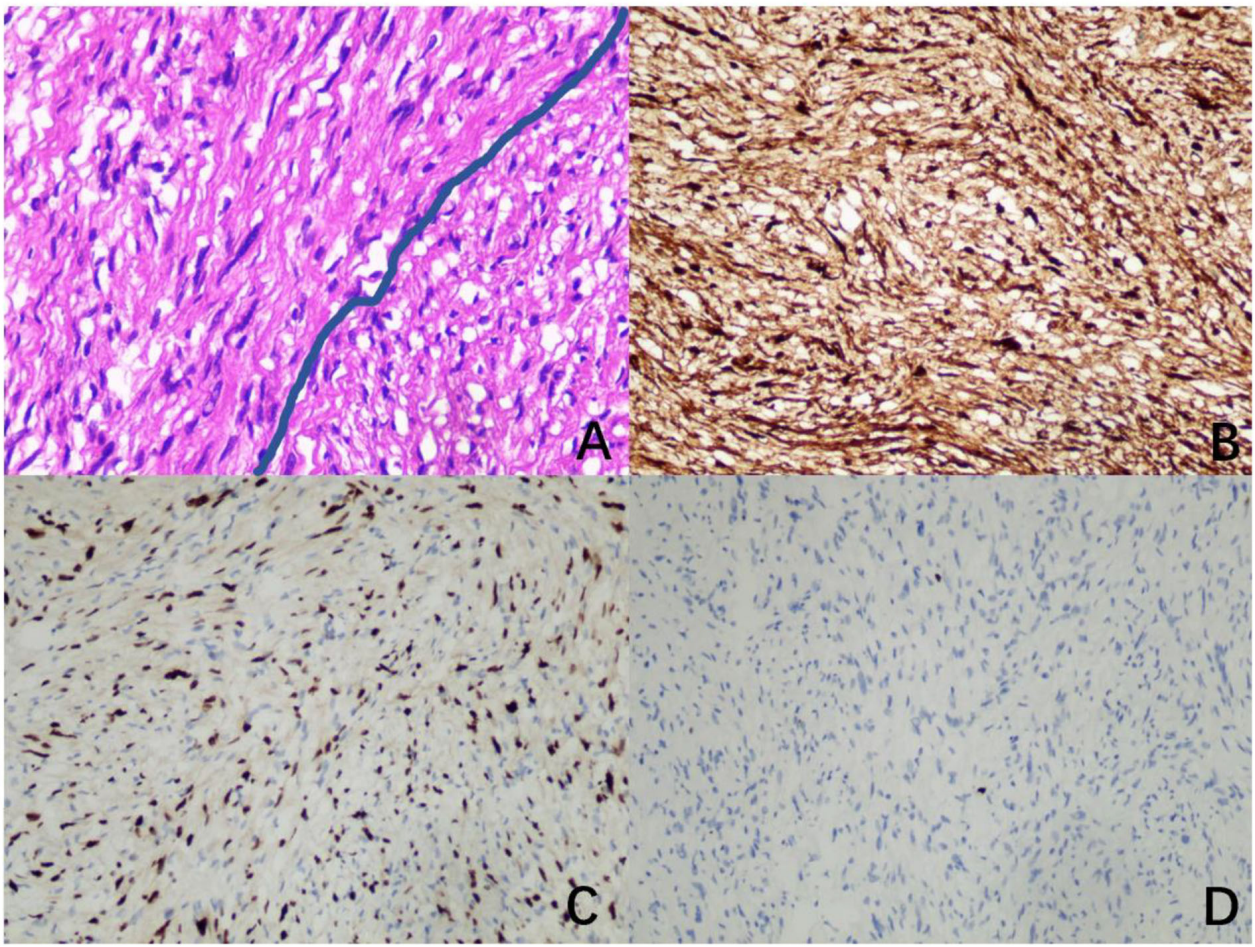
Image of pathology. **(A)** A biphasic pattern of Antoni A and Antoni B (hematoxylin and eosin, × 400). **(B)** Diffusely and strongly positive immunoreactivity for S100 (immunohistochemistry for S100, × 200). **(C)** Positive immunoreactivity for SOX10 (immunohistochemistry for SOX10, × 200). **(D)** Less than 1% positive immunoreactivity for KI67 (immunohistochemistry for KI67, × 200).

The mass had a biphasic pattern of Antoni A (palisading spindle tumor cells are arranged vertically and tightly) and Antoni B (haphazardly spindle and multipolar cells are arranged loosely as a reticular structure) under a microscope. Immunohistochemical analysis showed that the neural marker (S100 protein) was firmly and positively stained, which was specific for the diagnosis. The nuclei of those cells showed positive immunoreactivity for SOX10 and less than 1% of the nuclei showed positive immunoreactivity for KI67 (Figure 4).

## 3. Discussion

Schwannoma arises from Schwann cells and are known to originate from the sensory branches of the ophthalmic division of the trigeminal nerve in an orbit (3). Schwannomas occur in middle age and are usually rare in children with the absence of neurofibromatosis (4). We discussed a 12-year-old boy who had a dumbbell-shaped schwannoma, and most of the lesions were found in the left orbit with a small portion in the left cavernous sinus. Orbital schwannomas are usually oval or round, but those extending into the cranial cavity from the superior orbital fissure are usually dumbbell-shaped (5). Therefore, we speculated that it was a unique case of an orbital schwannoma in children extending into the left cavernous sinus *via* the superior orbital fissure.

Schwannomas are always benign and well-encapsulated nerve sheath tumors, with an insidious and slow growth pattern (6). There is no difference between genders or between the right or left eyes (3, 7, 8). Schwannomas have non-specific clinical presentations, and their primary clinical symptom is painless exophthalmos (8). They usually did not affect extraocular movements or vision unless it was big enough to compress the optic nerve, which might cause visual impairment, double vision, abnormal sensations, pain, eyelid swelling, and even vision loss (2, 9).

Microscopically, most orbital schwannomas exhibit a histopathologic feature with a biphasic morphology, including Antoni A and Antoni B patterns (10). Due to this situation, the tumor on MRI has a different degree of heterogeneity, which correlates with the dark signal of Antoni B and the bright signal of Antoni A on T1WI. However, the diagnosis of schwannomas *via* clinical symptoms and imaging evaluation is tricky because of the variable presentation and location (11). According to the literature, orbital schwannomas produce a hypointense signal on T1WI and a hyperintense signal on T2WI, which may be homogeneously or heterogeneously enhanced (12). CT is often the first investigation obtained, which is helpful in depicting adjacent bone destruction and calcification (13).

We conducted keyword searches in the PubMed database over the past decade, and the sets of keywords used were associated with orbital schwannoma. According to previous related rare case reports, we summarized the common location of orbital schwannomas, which was an intraocular, orbital, retro-orbital, infraorbital, trigeminal, vagal, or trochlear mass. In part, we also found a low incidence of orbital schwannomas in children, and we believed that the rarity could be explained by the slow-growing nature of this type of tumor type in some patients presenting in early adulthood (14). Though a few cases involving cranial-orbital communication were reported in the literature, we found nearly no case completely similar to our patient, a 12-year-old boy having a cranial-orbital schwannoma with a dumbbell-like appearance at the same time. Thus, we have reported this rare and unique case to enrich the information about intracranial-orbital schwannoma in children and also suggest that clinicians and radiologists should sufficiently differentiate it from other orbital tumors.

In the orbital extraocular area, optic nerve glioma is the most common tumor in children (7), whose signal is similar to that of the brain parenchyma, as in our case. However, we can ensure that the tumor is located above the optic nerve through imaging findings, which led us to rule it out. In addition, another common optic nerve disease in children is myelin oligodendrocyte glycoprotein (MOG) antibody-associated optic neuritis, characterized by visual loss and pain with eye movement (15), which is not consistent with the clinical presentation of our case. The mass in our case appears to be dumbbell-shaped, well-defined, mildly heterogeneous, and strengthening, suggesting that it may be a benign tumor with a low incidence. Thus, we hypothesized that it was a hamartoma before surgery, which usually had a clear edge, had a mildly non-uniform internal signal, hypointense and hyperintense on T1WI and T2WI, respectively, and had a slightly uneven enhancement (16). Fortunately, hamartoma and schwannoma were all benign masses and had the same surgical method, which did not delay the treatment of this child due to our misdiagnosis.

Usually, the first choice for orbital schwannoma is gross total resection (8, 17, 18). No further radiotherapy or chemotherapy is required after surgery. According to our prior recognition, only a few cases of orbital schwannoma with recurrence had been reported (19). However, we still need to follow up with the patient for a long period of time.

## 4. Conclusion

Our case refers to a 12-year-old boy with intracranial-orbital schwannoma. Preoperative diagnosis is difficult due to its different degrees of heterogeneity. However, early assessment and prompt management are necessary to prevent the development of severe complications. We suggest that orbital schwannomas should be sufficiently differentiated from other orbital tumors in children, despite their exceedingly rare occurrence. Our case enriches the information about this disease, enhances our understanding of this tumor, and underscores the importance of complete surgical resection.

## Data availability statement

The raw data supporting the conclusions of this article will be made available by the authors, without undue reservation.

## Ethics statement

Written informed consent was obtained from the minor's legal guardian, for the publication of any potentially identifiable images or data included in this article.

## Author contributions

YS, QX, AC, and SC performed the material preparation, image collection, and analysis. YS wrote the first draft of the manuscript. QX supervised the initial draft and critically revised the manuscript. SC and AC analyzed the case report and critically revised the manuscript. MQ corrected the grammar in the manuscript. All authors contributed to the case report conception and have read and approved the final manuscript.
